# Genome-wide identification, molecular evolution and expression analysis of the *B-box* gene family in mung bean (*Vigna radiata* L.)

**DOI:** 10.1186/s12870-024-05236-9

**Published:** 2024-06-12

**Authors:** Lili Yin, Ruigang Wu, Ruilan An, Yaxin Feng, Yaqi Qiu, Meiling Zhang

**Affiliations:** 1 College of Agronomy and Life Sciences, Shanxi Datong University, Datong, 037009 People’s Republic of China; 2 School of Landscape and Ecological Engineering, Hebei University of Engineering, Handan, 056038 People’s Republic of China; 3 Institute of Forestry and Pomology, Beijing Academy of Agriculture and Forestry Sciences, Beijing, 100093 People’s Republic of China

**Keywords:** Mung bean, *B-box* gene family, Evolution, Abiotic stresses, Expression profiles

## Abstract

**Background:**

Mung bean (*Vigna radiata* L.) is an important warm-season grain legume. Adaptation to extreme environmental conditions, supported by evolution, makes mung bean a rich gene pool for stress tolerance traits. The exploration of resistance genes will provide important genetic resources and a theoretical basis for strengthening mung bean breeding. B-box (BBX) proteins play a major role in developmental processes and stress responses. However, the identification and analysis of the mung bean *BBX* gene family are still lacking.

**Results:**

In this study, 23 *VrBBX* genes were identified through comprehensive bioinformatics analysis and named based on their physical locations on chromosomes. All the VrBBXs were divided into five groups based on their phylogenetic relationships, the number of B-box they contained and whether there was an additional CONSTANS, CO-like and TOC1 (CCT) domain. Homology and collinearity analysis indicated that the *BBX* genes in mung bean and other species had undergone a relatively conservative evolution. Gene duplication analysis showed that only chromosomal segmental duplication contributed to the expansion of *VrBBX* genes and that most of the duplicated gene pairs experienced purifying selection pressure during evolution. Gene structure and motif analysis revealed that *VrBBX* genes clustered in the same group shared similar structural characteristics. An analysis of *cis*-acting elements indicated that elements related to stress and hormone responses were prevalent in the promoters of most *VrBBXs*. The RNA-seq data analysis and qRT-PCR of nine *VrBBX* genes demonstrated that *VrBBX* genes may play a role in response to environmental stress. Moreover, *VrBBX5*, *VrBBX10* and *VrBBX12* are important candidate genes for plant stress response.

**Conclusions:**

In this study, we systematically analyzed the genomic characteristics and expression patterns of the *BBX* gene family under ABA, PEG and NaCl treatments. The results will help us better understand the complexity of the *BBX* gene family and provide valuable information for future functional characteristics of specific genes in this family.

**Supplementary Information:**

The online version contains supplementary material available at 10.1186/s12870-024-05236-9.

## Background

Zinc finger proteins are among the transcription factors that play important roles in developmental processes and stress responses [1]. The *B-box* (*BBX*) gene family belongs to the zinc finger protein. The BBX proteins have one or two B-box domains at the N-terminus of proteins, and some have a conserved C-terminal CONSTANS, CO-like and TOC1 (CCT) domain. Based on the distance between the zinc-binding residues and their consensus sequence, the BBX domain is divided into two categories: B-box1 (B1) and B-box2 (B2) [2]. Both the BBX and CCT domains have important functions. The BBX domain plays an important role in modulating protein‒protein interactions due to the inclusion of conserved cysteine and histidine [3, 4]. The CCT domain is important for transcriptional regulation and nuclear transport [5].

In *Arabidopsis*, 32 *BBX* genes are divided into five groups according to the number of B-box domains and whether the protein contains a CCT domain [3]. The first characterized *BBX* gene in *Arabidopsis* was *CONSTANS* (*CO*)/*AtBBX1*, which is a key activator of flowering under long inductive days. CO acts as a transcription factor to directly bind the promoter of *Flowering Locus T* (*FT*), ultimately triggering the expression of *FT* and initiating flowering [6]. The CO-FT module is highly conserved in regulating photoperiod flowering in various plants, such as rice, corn, tomato and sunflower [7]. In addition, other members of the BBX family have been reported to mediate flowering, including *AtBBX4*, *AtBBX7*, *AtBBX21* and *AtBBX32* [8–10]. Extensive studies have reported that BBX proteins act as key factors in the regulation of early photomorphogenesis. Some BBXs, such as *BBX11*, *BBX21*, *BBX22*, and *BBX23*, are positive regulators of photomorphogenesis [11–13], while others, including *BBX19*,*BBX24*, *BBX25*, *BBX28*, *BBX29*, *BBX30*, *BBX31* and *BBX32*, repress photomorphogenesis in response to a wide range of light signals [7, 14–16]. For example, BBX28 negatively regulates photomorphogenesis by inhibiting the activity of transcription factor HY5 and undergoes COP1-mediated degradation [16]. Several *BBX* genes also have functions in shade avoidance through mediating cell elongation [17].

An increasing number of studies have shown that BBX proteins play critical roles in abiotic stress responses. In *Arabidopsis*, AtBBX24 was initially isolated as a salt-tolerant protein (STO) conferring salt tolerance in yeast [18]. *STO* overexpression enhances root growth under high salinity conditions, indicating that *STO* is involved in the salt-stress response [19]. *AtBBX21*/*STH2* is a homolog of *STO*. Molecular lesions of *STH2* resulted in reduced stomatal aperture and water loss under abscisic acid (ABA) and sodium chloride (NaCl) treatments, indicating that *sth2* mutants are sensitive to ABA and salt stress. In addition, BBX21 negatively regulates *ABI5* expression by interfering with the binding of HY5 to the *ABI5* promoter, contributing to a decelerated ABA response. Therefore, *STH2* negatively regulates ABA-mediated dehydration tolerance [20]. The heterologous expression of *AtBBX29* in sugarcane enhances drought tolerance and delays senescence under water-deficit conditions by maintaining photosynthesis and enhancing antioxidant and osmolyte capacity [21]. In *Chrysanthemum*, CmBBX19 interacts with CmABF3 to repress the ABA response, resulting in reduced drought tolerance [22], while CmBBX22 delays drought-induced leaf senescence by negatively regulating the expression of *ABF4* and upregulating the expression of *ABI3* and *ABI5* [23]. CmBBX24 improves freezing and drought stress tolerance by negatively regulating gibberellin biosynthesis and positively regulating genes associated with compatible solutes and carbohydrate metabolism [24]. *MdBBX10* overexpression in *Arabidopsis* significantly enhances tolerance to abiotic stresses, with a higher germination ratio and longer root length. In addition, *MdBBX10* overexpression can enhance a plant’s ability to scavenge reactive oxygen species (ROS) under stress, which is correlated with the expression of ROS-scavenging genes such as superoxide dismutase (*SOD*), ascorbate peroxidase (*APX*) and glutathione s-transferase (*GST*) [25]. *MdBBX1* transgenic plants display enhanced tolerance and have a higher survival rate after salt and drought treatments [26]. Overexpression of Ginkgo *BBX25* improves salt tolerance in transgenic Populus [27]. *CpBBX19* (*Chimonanthus praecox*) confers salt and drought tolerance in *Arabidopsis* [28]. Similarly, overexpression of *IbBBX24* enhances salt and drought tolerance by scavenging ROS in sweet potato [29]. In addition to their roles in photomorphogenesis, flowering, shade avoidance and stress responses, increased studies have revealed that BBX proteins also play important roles in the circadian rhythm, senescence, and anthocyanin biosynthesis [7].

*BBX* genes have been systematically identified and analyzed in several species, e.g., there are 30 *BBX* genes in rice [4], 29 in tomato [30], 25 in pear [31], 64 in apple [32] and 24 in grapevine [33]. However, thus far, *BBX* genes have not been well studied in mung bean. Mung bean belongs to the subfamily *Papilionoid* of Fabaceae and is widely cultivated in tropical and subtropical regions. As a functional raw food material, mung bean is rich in nutrients, including high-quality proteins, carbohydrates, dietary fibers and bioactive substances [34]. Because of its nutritional value and broad adaptation, attention has been focused on developing resistance genes to enhance mung bean breeding. With the availability of the mung bean genome sequence [35], the *BBX* gene family in mung bean could be systematically studied. Here, we analyzed the phylogenetic evolutionary relationships, chromosome localization, motifs, genetic structure, *cis*-acting elements and expression profiles under polyethylene glycol (PEG), NaCl and ABA treatments. This study provides a new understanding of the evolutionary mechanism of *BBX* genes and lays a foundation for future investigations of the biological functions of *BBX* genes in mung bean.

## Results

### Identification of *BBX* genes in mung bean

Taking advantage of the availability of the complete genome assembly of mung bean, we initially identified 42 non-redundant *BBX* genes in mung bean using *Arabidopsis* BBX proteins as queries. Then, the Hidden Markov Model (HMM) profile of the B-box was used to search for putative *BBX* genes, and 19 candidate *BBX* genes were found. The resulting sequences were further confirmed as BBX members by identifying the presence of the B-box domain. As a result, 23 *VrBBX* genes were confirmed in mung bean and named *VrBBX1* to *VrBBX23* based on their physical locations on the chromosomes. A total of 17 *VrBBX* genes were unevenly distributed on 9 of 11 chromosomes, and the other 6 genes (*VrBBX18*, -*19*, -*20*, -*21*, -*22* and -*23*) have not yet been assembled to any chromosome (Additional file 1). Table 1 shows details on *VrBBXs*, including gene names, accession number, chromosome location, coding sequence length (CDS), protein length, molecular weight (MW) and theoretical isoelectric point (pI). The divergent lengths of 23 VrBBX proteins resulted in diverse MWs and pIs, indicating that the VrBBXs varied greatly in sequence and physicochemical properties.

**Table 1:** Detailed information of *VrBBX* genes in mung bean

Gene names	Accession number	Chromosome: location	CDS/bp	Size/AA	MW (kDa)	pI
*VrBBX1*	Vradi01g04550	1:6969585:6973203	864	287	32.31	6.95
*VrBBX2*	Vradi01g07820	1:12516640:12519285	1098	365	40.94	5.26
*VrBBX3*	Vradi02g04040	2:3764677:3767502	750	249	27.29	4.91
*VrBBX4*	Vradi02g11390	2:21437315:21442108	891	296	32.46	5.62
*VrBBX5*	Vradi03g05670	3:7206384:7210968	1035	344	38.35	6.47
*VrBBX6*	Vradi04g04920	4:11016354:11020161	1119	372	41.85	7.00
*VrBBX7*	Vradi05g11170	5:20065480:20066834	876	291	31.86	8.19
*VrBBX8*	Vradi05g22760	5:34939846:34944202	1332	443	49.03	5.84
*VrBBX9*	Vradi06g07140	6:10071923:10080664	1002	333	36.49	8.19
*VrBBX10*	Vradi06g16830	6:37220123:37221509	729	242	26.84	7.11
*VrBBX11*	Vradi07g01030	7:2077743:2079449	615	204	22.57	8.49
*VrBBX12*	Vradi07g21950	7:44801181:44803116	1119	372	40.40	6.11
*VrBBX13*	Vradi07g27680	7:51183172:51185080	1110	369	41.40	5.64
*VrBBX14*	Vradi08g09990	8:27297889:27300082	942	313	35.65	7.25
*VrBBX15*	Vradi08g20920	8:43034584:43041216	1482	493	54.87	4.84
*VrBBX16*	Vradi08g21370	8:43588019:43589633	876	291	32.45	8.78
*VrBBX17*	Vradi11g06190	11:6151451:6153702	558	185	20.37	6.54
*VrBBX18*	Vradi0070s00170	scaffold_70:586589:588154	855	284	31.11	4.34
*VrBBX19*	Vradi0153s00350	scaffold_153:194458:195661	738	245	26.41	9.17
*VrBBX20*	Vradi0169s00070	scaffold_169:696687:698671	1113	370	42.03	9.22
*VrBBX21*	Vradi0332s00150	scaffold_332:165731:168217	726	241	26.63	4.74
*VrBBX22*	Vradi0364s00080	scaffold_364:145806:148452	768	255	27.57	5.21
*VrBBX23*	Vradi0393s00030	scaffold_393:136784:139426	666	221	25.21	7.09

### Conserved domain analysis of VrBBXs

**Fig. 1:**
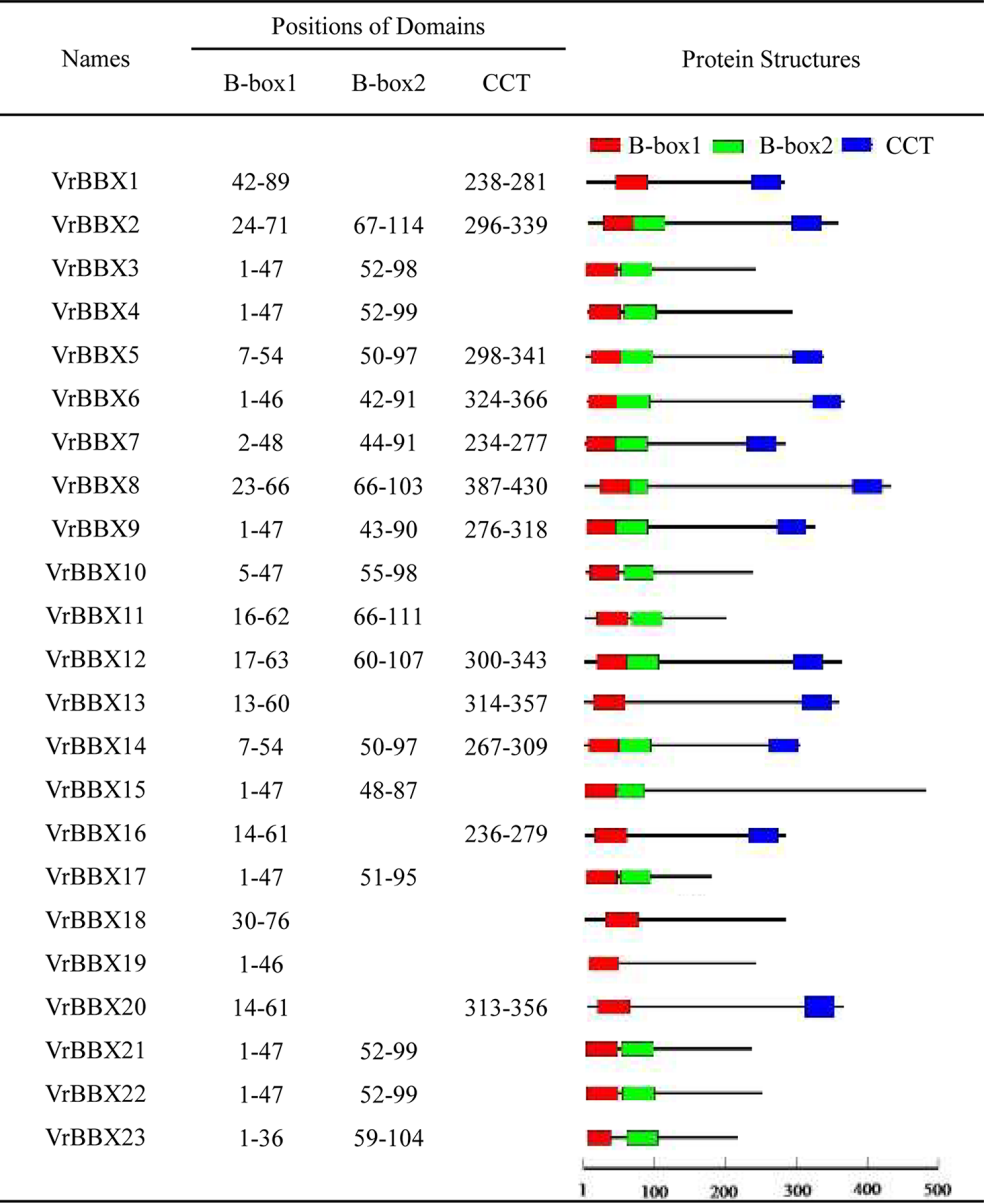
The diagrams of conserved domains for the VrBBX proteins. The length of each protein sequence is represented by the black bar. The colored boxes refer to the conserved domains: red box, B-box1 domain; green box, B-box2 domain; blue box, CCT domain. The sequence length of each protein is represented at the bottom and the scale bar represents 100 amino acids

**Fig. 2:**
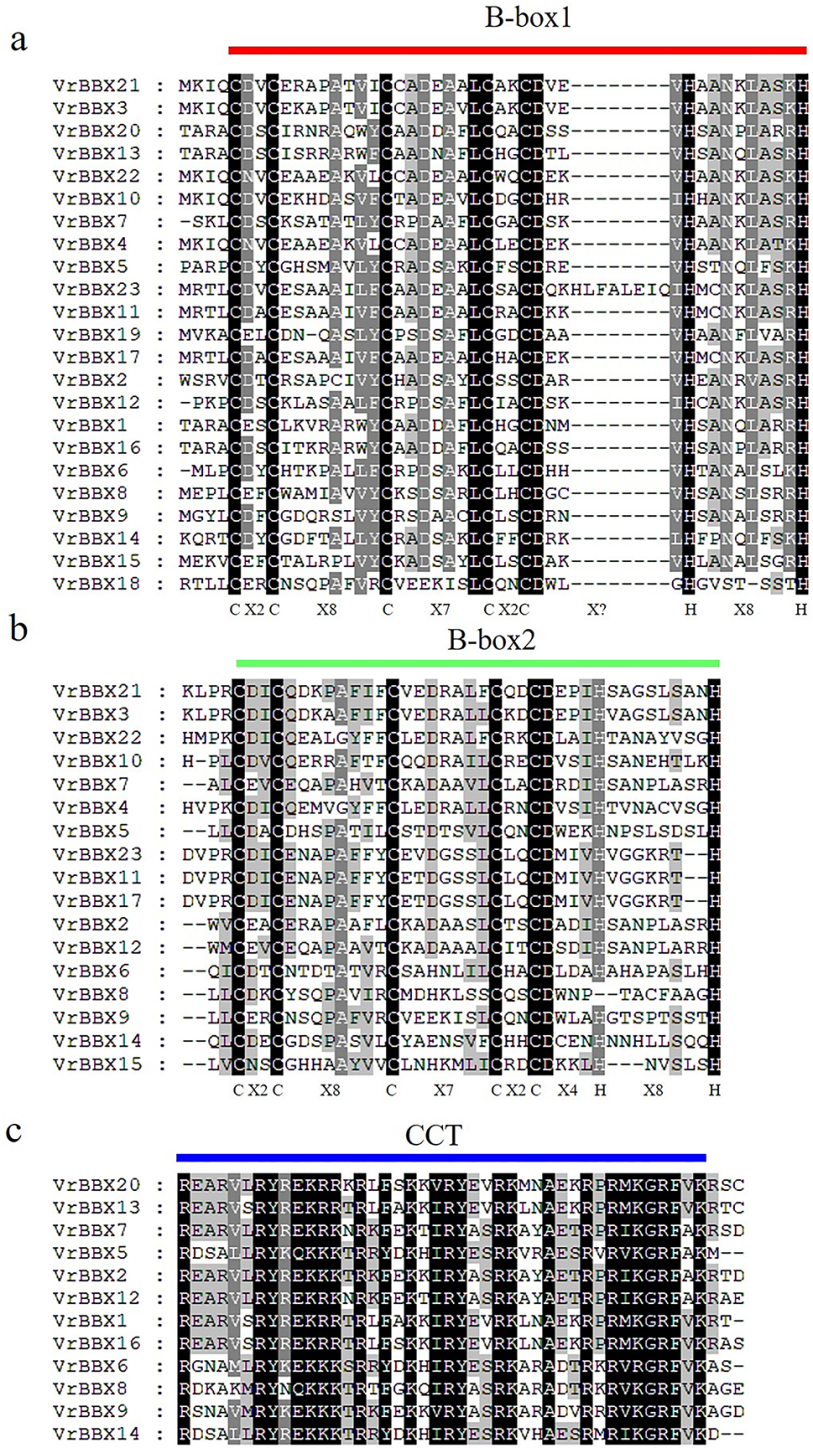
Multiple sequence alignments of the B-box1 (**a**), B-box2 (**b**) and CCT (**c**) domains. The identical amino acids, conserved amino acids and similar amino acid residues are shaded in black, charcoal gray and gray, respectively

**Fig. 3:**
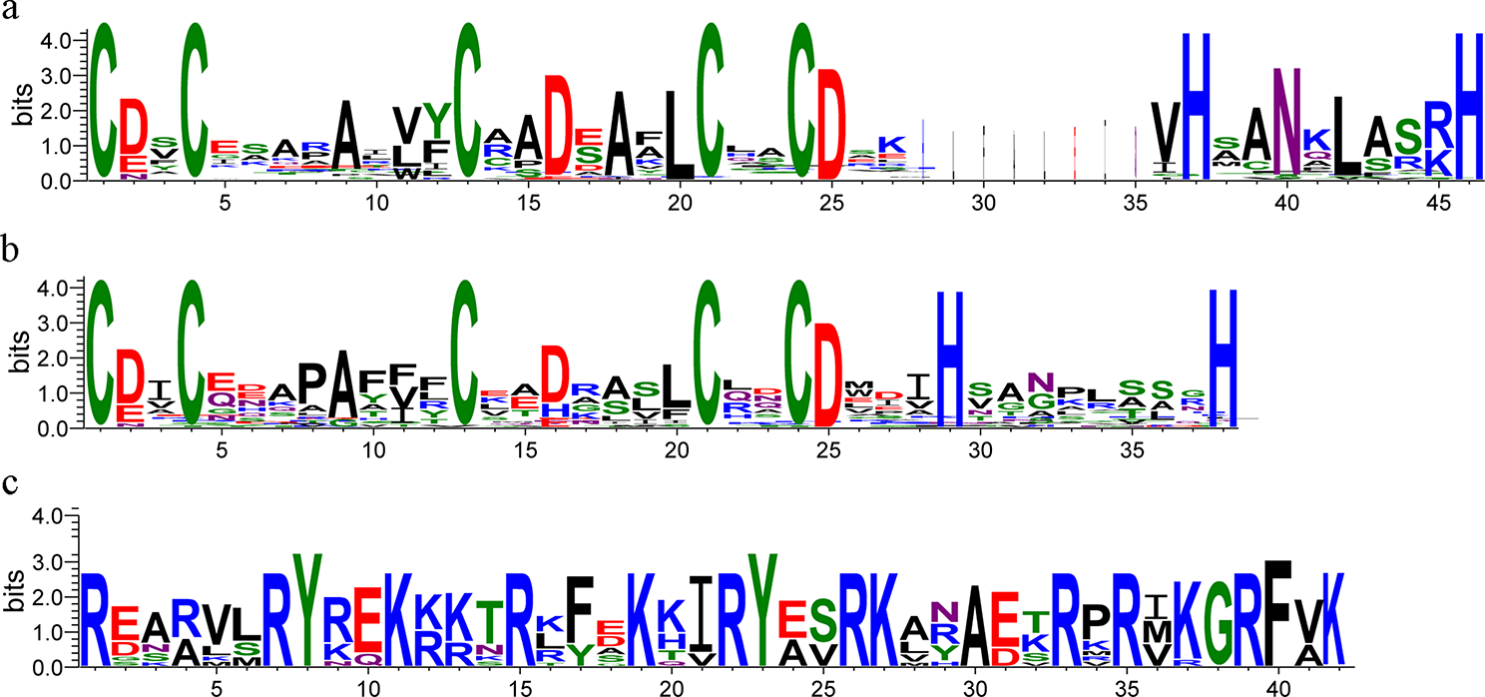
The conserved domains in the VrBBX proteins. Logos of the protein alignment of the B-box1 **(a**), B-box2 (**b**) and CCT domains (**c**) are shown. The x-axis represents the conservative sequences of the domains. The height of each letter represents the conservation of each residue across all proteins. The y-axis is the scale of the relative entropy that reflects the conservation rate of each amino acid

Among the 23 VrBBX proteins, eight VrBBXs had two conserved B-box domains and a CCT domain, and nine had two B-box domains. Four VrBBXs consisted of one B-box domain and one CCT domain, while two VrBBXs had only one B-box domain (Fig. 1). The sequence alignment of VrBBX proteins indicated that B-box1, B-box2 and CCT domains had similar conserved amino acid residues and that B-box1 was more conserved than B-box2 (Fig. 2). The conserved B-box1 sequence was CDXCXXXXAXVYCXADXAXLCXXCDXXVHXANXLASRH (where “X” represents any amino acid), and the B-box2 sequence was CDICEXXPAFVXCXXDXXLLCXXCDXXIHXXXXXSXXH. The conserved CCT sequence was REARVLRYREKRKTRKFXKXIRYESRKXXAETRPRIKGRFVK. However, B-box1 and B-box2 retained the same topology, with the form of CX2CX8CX7CX2CX? HX8H (where “?” represents multiple amino acids) (Fig. 2). Figure 3 shows the logos of B-box1, B-box2 and CCT domains.

### Phylogenetic analysis of the mung bean *BBX* gene family

**Fig. 4:**
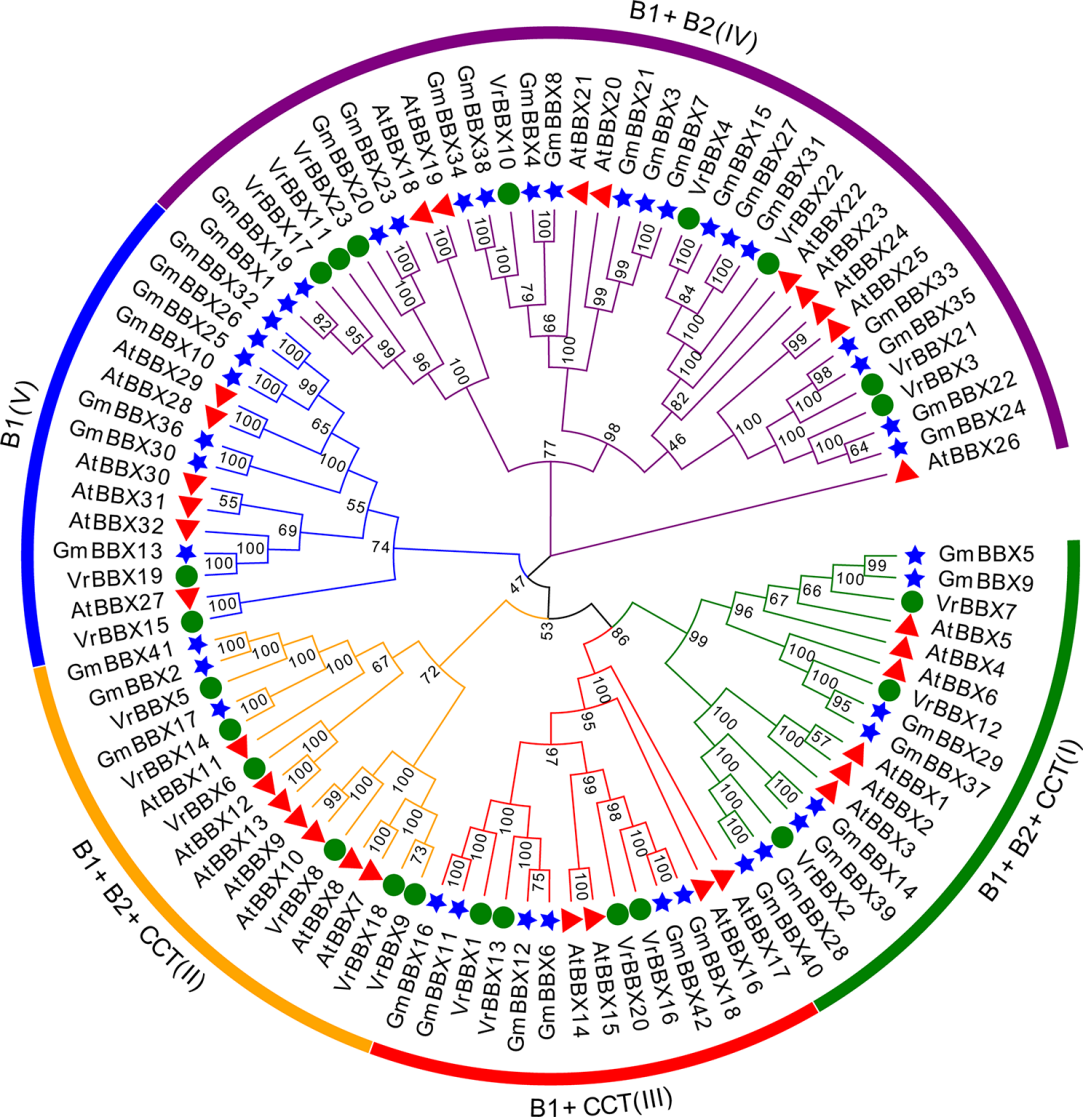
Phylogenetic tree of BBXs. The full-length amino acid sequences of BBX in mung bean, soybean and *Arabidopsis* are used for Neighbor-joining (NJ) phylogeny reconstruction with 1000 bootstrap replicates and the bootstrap values are indicated at each node. The green circles, red triangles and blue stars represent VrBBXs, AtBBXs and GmBBXs, respectively. The BBX proteins are divided into five groups (I-V) with different colors

To explore the phylogenetic relationships of the BBX family in mung bean, *Arabidopsis* and soybean, a phylogenetic tree was constructed based on the 97 aligned BBX protein sequences (Additional file 2). All 97 BBXs were divided into five groups (I, II, III, IV and V) (Fig. 4). Members in groups I and II contained two B-boxes and an additional CCT domain, but the amino acid sequence of the B-box2 domain was more conserved in group I than in group II (Additional file 3). Group III BBX members had a B-box1 domain and a CCT domain, and group IV BBX members contained B-box1 and B-box2 domains. Group V BBX members had only a B-box1 domain (Fig. 4). However, some of the BBXs did not fit the presented classification scheme. For example, the members of group II contained two B-boxes and one CCT domain, except for VrBBX18, which only had one B-box domain. VrBBX15 contained the B-box1 and B-box2 domains, suggesting that it belonged to group IV but was clustered in group V in the phylogenetic tree (Figs. 1 and 4). In addition, each group included BBX members from mung bean, *Arabidopsis* and soybean, indicating that the *BBX* genes originated before the differentiation of mung bean, *Arabidopsis* and soybean.

### Collinearity analysis of *BBX* genes in mung bean, rice, *Arabidopsis* and soybean

**Fig. 5:**
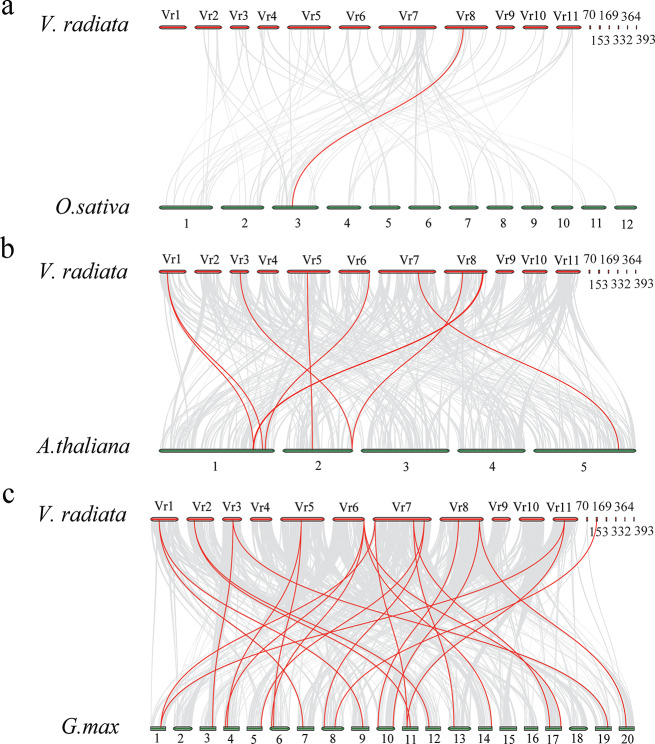
Synteny analysis of *BBX* genes between mung bean and monocotyledonous rice (**a**), monocotyledonous *Arabidopsis* (**b**) and soybean (**c**). *V. radiata, O. sativa, A. thaliana* and *G. max* represent *Vigna radiata*, *Oryza sativa, Arabidopsis thaliana* and *Glycine max*, respectively. Gray lines in the background are the duplicated gene pairs between mung bean and other plant genomes, while the red lines indicate the syntenic *BBX* gene pairs. The colored bars represent chromosomes or scaffolds, and their numbering is displayed at the top or bottom of the colored bars

To understand the gene duplication mechanisms of *BBXs*, we constructed three comparative syntenic maps of mung bean associated with three representative species, comprising a monocot (rice) and two dicots (*Arabidopsis* and soybean). The *VrBBX* genes exhibited the highest homology with the *BBX* genes of soybean (24), followed by *Arabidopsis* (9) and rice (1) (Fig. 5, Additional file 4). Both *VrBBX14* and *VrBBX19* shared collinearity with only one soybean *BBX* gene. Some *VrBBX* genes (*VrBBX1, -3, -5, -7, -11, -12, -13, -16* and *-17*) were associated with two syntenic gene pairs, and *VrBBX10* was associated with four syntenic gene pairs. No collinear segments of *VrBBX2, -4, -6, -8, -9, -15, -18, -20, -21, -22* and *-23* were found in the genomes of mung bean and soybean (Additional file 4). Moreover, *VrBBX14* was collinear with the genomes of rice, *Arabidopsis* and soybean. *VrBBX1, -5, -7, -10, -12* and *-16* were collinear with the genomes of *Arabidopsis* and soybean (Fig. 5, Additional file 4).

### Gene duplication of *VrBBX* genes in mung bean

**Fig. 6:**
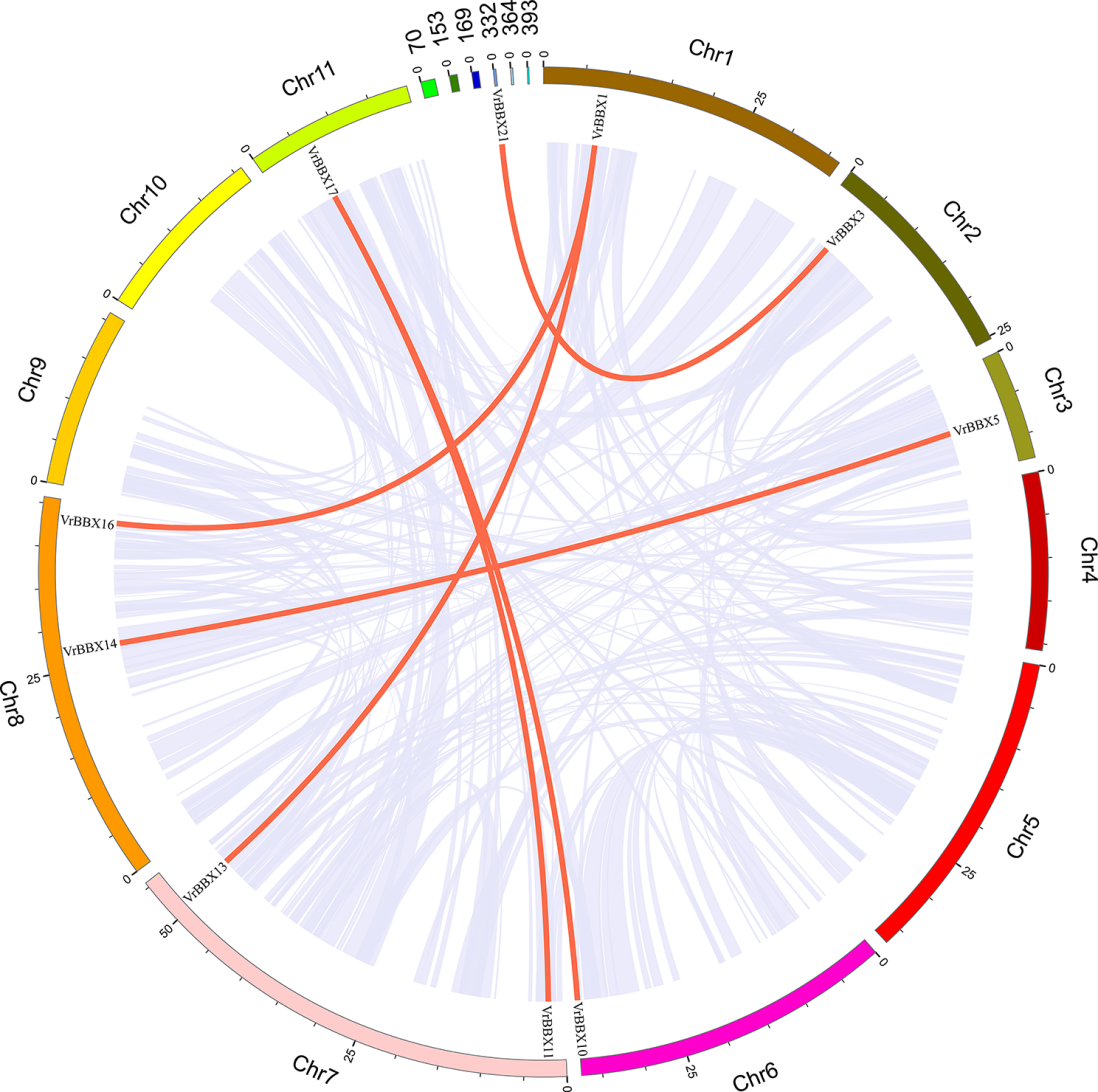
Synteny analysis of *VrBBX* genes on mung bean chromosomes. The light purple lines in the background indicate all synteny blocks in the mung bean genome between each chromosome, and the thick red lines indicate duplicate *VrBBX* gene pairs. The colored boxes indicate the different chromosomes or scaffolds and their numbering is shown on the outside of boxes. The scale bar marked on the chromosome is the length of the chromosome (Mb)

To better elucidate the expansion patterns of *VrBBX* genes, synteny was also used to analyze mung bean *BBX* gene duplication. Six segmental duplication events were identified with ten *VrBBX* genes, which were located on duplicated segments on chromosomes 1, 2, 3, 6, 7, 8 and 11 and scaffold_364 (Fig. 6). No tandem duplication pairs were observed in our results. Subsequently, the Ka/Ks ratio of the duplicated gene pairs was calculated to determine the selection pressure. All Ka/Ks ratios were less than 1, except for that of gene pair *VrBBX1*/*VrBBX13*, indicating that most were under purifying selection during evolution. The divergence time between collinear gene pairs varied from 19.841 to 33.910 million years (Mya) (Table 2).

**Table 2:** Ka/Ks analysis for duplicated gene pairs of *BBXs* in mung bean

Duplicated Gene 1	Duplicated Gene 2	Type of duplication	Ka	Ks	Ka/Ks	Purifying Selection	Divergence-Time (MYA)
*VrBBX10*	*VrBBX17*	segmental	0.99455	1.01731	0.97763	Yes	33.910
*VrBBX3*	*VrBBX21*	segmental	0.09726	0.59522	0.16341	Yes	19.841
*VrBBX11*	*VrBBX17*	segmental	0.08754	0.61510	0.14232	Yes	20.503
*VrBBX1*	*VrBBX16*	segmental	0.75151	0.78070	0.96261	Yes	26.023
*VrBBX1*	*VrBBX13*	segmental	0.69935	0.64817	1.07897	No	21.606
*VrBBX5*	*VrBBX14*	segmental	0.38052	0.81999	0.46405	Yes	27.333

### Gene structure and conserved motif analysis of the *BBX* gene family in mung bean

**Fig. 7:**
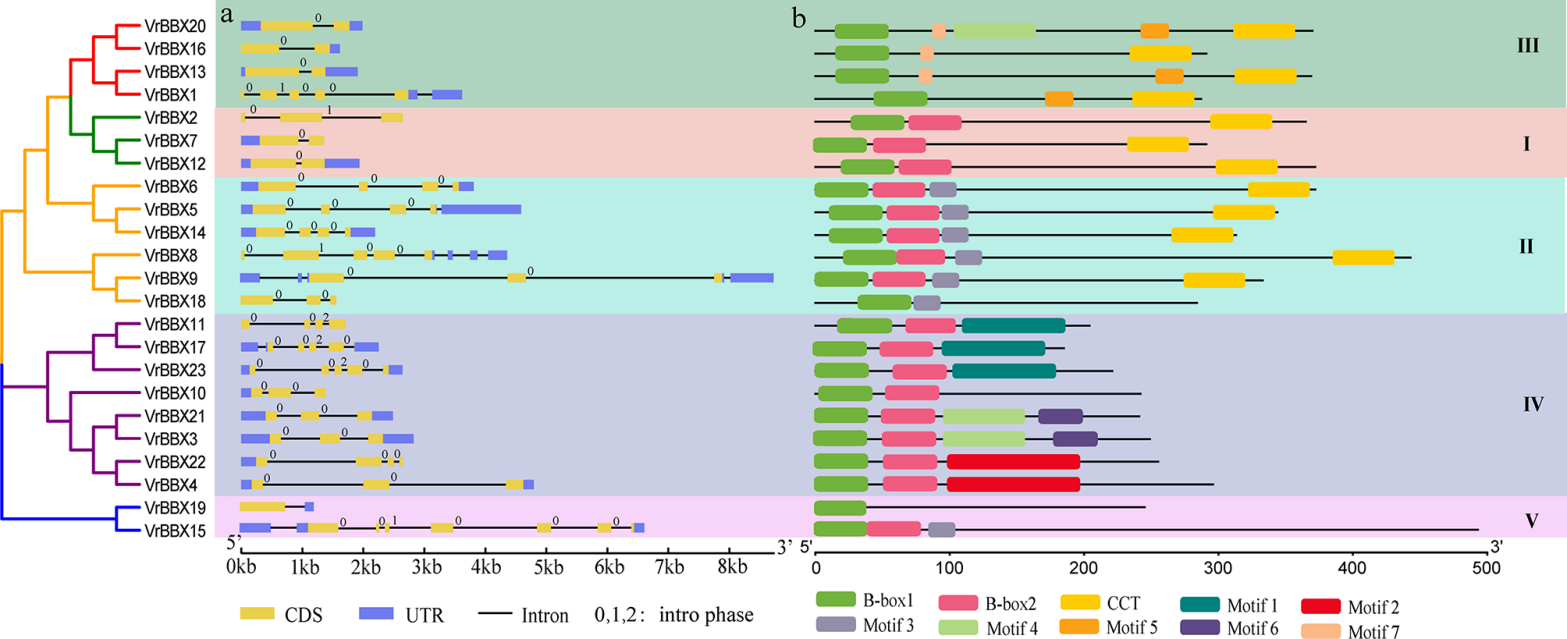
Phylogenetic tree, exon–intron structure and motif analysis of the *BBX* gene family in mung bean. **a** Structure analysis of *VrBBX* genes. Exons and introns are represented by yellow boxes and black lines, respectively. The untranslated regions (UTRs) are represented by blue boxes. The numbers (0, 1 and 2) indicate the intron phase. **b** Conserved motif analysis of VrBBX proteins. Each motif is represented by a number in a colored box

We mapped the exon‒intron map of *VrBBX* genes, and the number of exons ranged from 1 to 7. The *VrBBX* genes of groups I and III had two exons, except for *VrBBX1* (five exons) and *VrBBX2* (three exons). In groups II and IV, the majority of *BBX* genes contained three or four exons, with the exceptions of *VrBBX8* and *VrBBX23* (five exons). In group V, *VrBBX19* contained the fewest exons (one), and *VrBBX15* had the most exons (seven). (Fig. 7a). In addition, the intron patterns formed by relative position and phase in each group were highly conserved (Fig. 7a).

To further explore the potential function of VrBBXs, we analyzed the conserved motifs and detected seven novel motifs (motifs 1–7) in addition to the B1, B2 and CCT domains (Fig. 7b, Additional file 5). Some conserved motifs were restricted to specific groups. For instance, motifs 5 and 7 were detected only in group III, and motifs 1, 2 and 6 were found only in group IV (Fig. 7b), indicating a functional difference between groups III and IV. The results also showed different motifs within the same group, suggesting that VrBBXs within the same group performed different functions. For example, VrBBX1 in group III lacked motif 7, whereas the other three VrBBXs (VrBBX13, -16 and -20) in group III contained motif 7 (Fig. 7b). This phenomenon was also observed in the other groups. VrBBX19 in group V did not contain these seven motifs.

### *Cis*-acting element analysis in the promoters of *VrBBX* genes

**Fig. 8:**
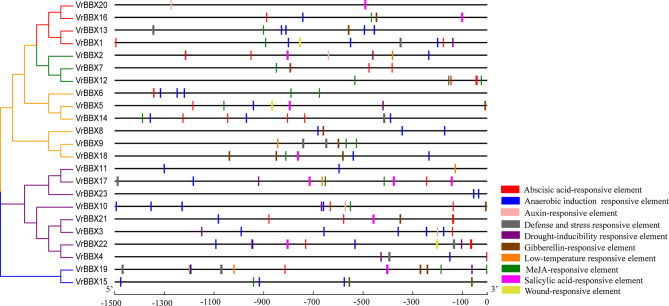
Predicted cis-elements in the promoter regions of *VrBBX* genes. The scale bar at the bottom indicates the length of promoter sequence (− 1500 bp). The *cis*-acting elements were represented by colored boxes

In the present study, we analyzed the *cis*-acting elements, including stress response and hormone-related elements, in the − 1.5 kb promoter region of the *VrBBX* genes. Anaerobic induction-responsive element, methyl jasmonate (MeJA)-responsive element and abscisic acid-responsive element were abundant in the promoter regions of *VrBBX* genes (Fig. 8, Additional file 6). Drought inducibility-responsive element was identified in the promoter of *VrBBX1, -2, -3, -4, -5, -10, -17, -19 and -22*, and the wound-responsive element was identified in the *VrBBX1, -5* and *-22* promoter regions. Low temperature-responsive element was found in the *VrBBX2, -9, -11, -17*, and *-19* promoter regions, and defense- and stress-responsive elements were found in the *VrBBX1, -4, -9, -13, -14, -17, -19* and *-22* promoter regions (Fig. 8, Additional file 6). These results indicate that some *VrBBX* genes might respond to various stresses and react collectively under the same stress. In addition, the promoter regions of *VrBBX* genes from the same group had similar *cis*-acting elements. For example, most of the group I *VrBBX* promoters had ABA- and MeJA- responsive elements, and most of the group III members contained anaerobic induction-responsive element and MeJA-responsive element (Fig. 8, Additional file 6). These results indicate that the same group of *VrBBX* genes may have similar functional mechanisms. In group III, only *VrBBX20* contained an auxin-responsive element, and only *VrBBX1* contained a wound-responsive element (Fig. 8, Additional file 6). This phenomenon was also found in other groups, indicating that the same group of *VrBBX* genes might have different mechanisms of action.

**Expression analysis of*VrBBX*genes**.

**Fig. 9:**
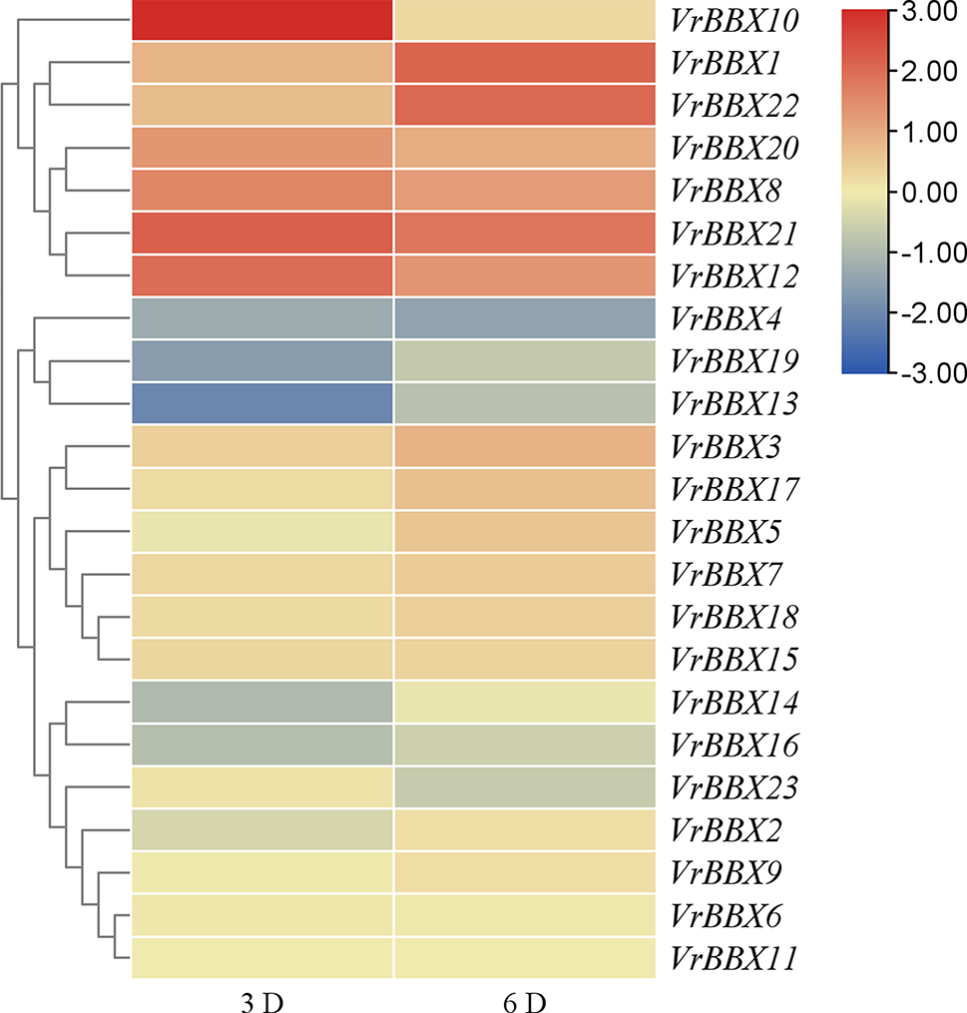
Heatmap expression profiles of *VrBBX* genes under drought stress. Red color indicates up-regulated gene expression, yellow color indicates no significant change in gene expression, and blue color indicates down-regulated gene expression under drought stress

For a better understanding of BBX genes involvement in abiotic stress, we analyzed the available RNA sequence data on the SRA database to get the expression of *VrBBX* genes. In the project, leave samples were taken after the drought stress treatment for 3 days and 6 days. Clustering analysis revealed clusters of genes (i) upregulated in response to drought stress (*VrBBX1*, -*8*, -*10*, -*12*, -*20*, -*21* and -*22*), (ii) no significant expression changes in response to drought stress (*VrBBX2*, -*3*, -*5*, -*6*, -*7*, -*9*, -*11*, -*14*, -*15*, -*16*, -*17*, -*18* and -*23*), (iii) downregulated in response to drought stress (*VrBBX4*, -*13* and -*19*) (Fig. 9).

**Fig. 10:**
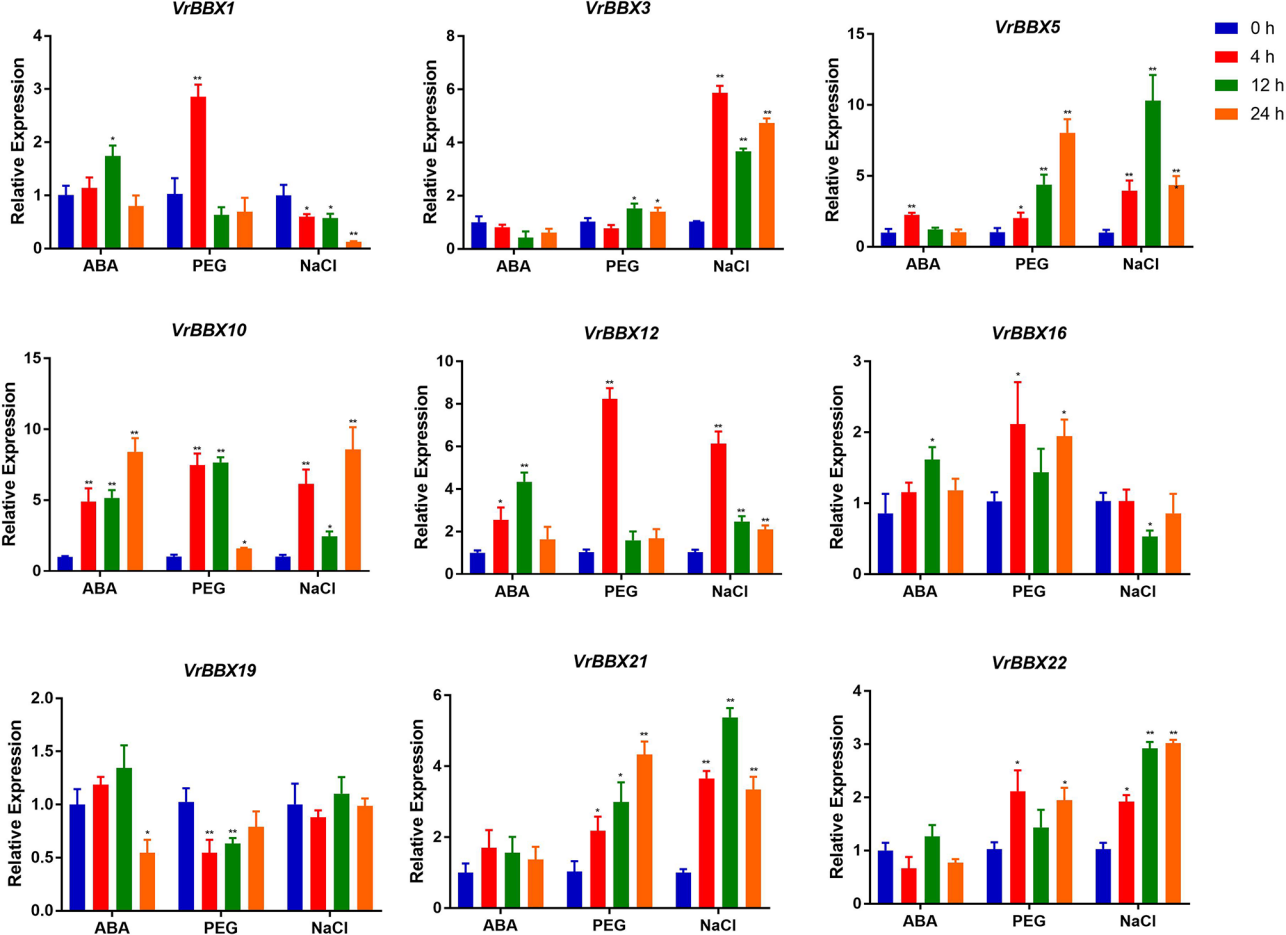
Expression profile of nine selected *VrBBX* genes in response to ABA (**a**), PEG (**b**) and NaCl (**c**) treatments. The mean value was calculated from three independent replicates. Vertical bars indicate the standard deviation. Asterisks indicate corresponding genes that were significantly upregulated or downregulated compared with the control (* *p* < 0.05; ** *p* < 0.01)

In *silico* expression analysis and cis-regulatory element analysis show that *BBX* genes may be involved in response to different abiotic stresses. Therefore, to predict the potential role of the *BBX* genes in mung bean, qRT-PCR was performed to estimate the transcript abundance of the *BBX* genes in leaf tissues under ABA, PEG and NaCl treatments. According to the homology of reported abiotic stress-related *BBX* genes and the presence of “defense and stress responsive element”, “drought-inducibility responsive element” or “abscisic acid-responsive element” in the promoters of *VrBBX* genes, nine *VrBBX* genes (*VrBBX12* from group I, *VrBBX5* from group II, *VrBBX1* and *VrBBX16* from group III, *VrBBX3*, *VrBBX10*, *VrBBX21* and *VrBBX22* from group IV, and *VrBBX19* from group V) were selected to study their expression profiles when treated with ABA, PEG and NaCl at 4, 12 and 24 h. Among them, *VrBBX3* and *VrBBX21* were orthologous to *AtBBX24*, and *VrBBX10* was orthologous to A*tBBX21*. *AtBBX24* was involved in salt-stress response, and *AtBBX21* mutants were sensitive to ABA and salt stress [19, 20]. The promoters of *VrBBX1*, *-19* and *-22* contained “defense and stress responsive elements.” All nine *VrBBX* gene promoters, except for *VrBBX12*, *-16* and *-21*, contained “drought-inducibility responsive element.” All nine *VrBBX* gene promoters had an “abscisic acid-responsive element”. The results showed that most of *VrBBX* genes were responsive to ABA, PEG and NaCl treatments. The expression profiles showed that *VrBBX5*, -*10* and -*12* were upregulated under all three treatments (Fig. 10). Among the upregulated genes, the relative expression level of *VrBBX10* showed the maximum fold change at 24 h under ABA treatment, and *VrBBX5* showed the maximum fold change at 12 h under NaCl stress. The relative expression levels of *VrBBX5* and *VrBBX12* were highest under PEG stress (Fig. 10). In case of ABA treatment, *VrBBX19* was found downregulated, while *VrBBX1*, *VrBBX5*, *VrBBX10*, *VrBBX12* and *VrBBX16* were found upregulated. There were no significant changes found in *VrBBX3*, *VrBBX21* and *VrBBX22* (Fig. 10). In case of drought stress, *VrBBX19* was found downregulated, while *VrBBX1*, *VrBBX3*, *VrBBX5*, *VrBBX10*, *VrBBX12, VrBBX16, VrBBX21* and *VrBBX22* were found upregulated (Fig. 10). In case of NaCl stress, *VrBBX1* and *VrBBX16* were found downregulated, while *VrBBX3*, *VrBBX5*, *VrBBX10*, *VrBBX12*, *VrBBX21* and *VrBBX12* were found upregulated. There was no significant change found in *VrBBX19* (Fig. 10).

## Discussion

BBX proteins are important regulators in mediating developmental processes and stress responses [36]. In the present study, we identified 23 *VrBBX* genes by performing a whole genome-wide analysis in mung bean. The number of *BBX* genes in mung bean was less than their orthologues in other species, such as *Arabidopsis* (32) [3] and rice (30) [4]. The genome size of mung bean is approximately 494–579 Mb, and 22,368 genes have been predicted [35]. Notably, the mung bean genome size is larger than those of rice (403 Mb) [37] and *Arabidopsis* (125 Mb) [38]. These results indicate that the BBX family members might not be directly related to the genome sizes of different plants [31]. Based on the phylogenetic relationships, the number of B-box they contain and whether there was an additional CCT, *VrBBX* genes were divided into five groups and clustered with the *BBXs* from other plant species, suggesting that they might have undergone similar evolutionary diversification [33]. The number of groups varied among plant species. There were 13, 4, 8 and 7 *BBXs* in *Arabidopsis* [3]; 7, 10, 10 and 3 *BBXs* in rice; and 9, 4, 8 and 2 *BBXs* in mung bean in groups I/II, III, IV and V, respectively [4]. These results suggest that BBX family members had a common ancestor and were independently expanded during speciation.

Previous research has shown that the amino acid sequence of B-box1 is different from that of B-box2, while B-box1 and B-box2 have the same topology [4, 31]. Our results showed that the B-box1 domain was more conserved than the B-box2 domain (Fig. 2). These results suggest that the early BBX proteins in green plants likely initially had one B-box domain and that the other B-box domain evolved by duplication events [2]. This assumption is supported by the evidence that most green algae have only a single B-box domain, and the first B-box duplication event occurred in *CrBBX1* of the green alga *Chlamydomonas* before the colonization of land plants [39]. The BBX proteins of groups I and II contained two B-boxes and an additional CCT domain. However, the amino acid sequence of the B-box2 domain in group I was more conserved than that in group II (Additional file 3), indicating that the evolutionary mechanism of B-box2 in groups I and II might be different. Previous research predicted that the BBX proteins of group II (B1 + B2 + CCT) were generated by adding a CCT domain at the C-terminus of group IV BBX proteins (B1 + B2). Then, the deletion of the B2 domain generated BBX proteins with a single B-box and CCT domain (group III). The duplication events of the B1 domain in group III BBX proteins may produce the B1 + B2 + CCT domain (group I). The early BBX proteins of group IV (B1 + B2) may be the ancestors of BBX members in group V (B1) after B2 domain deletion [2]. Therefore, the B-box2 domain in groups I and II differed in amino acid sequence, which is consistent with our research result. This indicates that the evolution of *VrBBX* in mung bean is consistent with the evolutionary model of previous studies.

To further clarify the evolutionary history of the BBX gene family, the synteny relationships of BBXs from monocots (rice) and dicots (mung bean, *Arabidopsis* and soybean) were systematically analyzed. *VrBBX14* had homologous gene pairs with *BBX* in *Oryza sativa* (1) (Fig. 5), implying that BBX family members had a common ancestor and that large-scale expansion of *BBXs* might have occurred after the division of monocots and dicots. Seven *VrBBX* genes (*VrBBX1, -5, -7, -10, -12, -14* and *-16*) had syntenic gene pairs in both *Arabidopsis* and soybean (Fig. 5), indicating that these *VrBBX* genes originated before the differentiation of mung bean, *Arabidopsis* and soybean. Five *VrBBX* genes (*VrBBX3, -11, -13, -17* and -*19*) might have originated before legume differentiation because of their collinear relationship between mung bean and soybean, while some *VrBBX* genes had no collinear segments (Fig. 5). Gene duplication plays a key role in generating novel genes during the process of plant evolution. There are two main patterns of gene replication in plants, segmental duplication and tandem duplication, which have been demonstrated to play an important role in the expansion of the gene family member [40]. Therefore, we investigated the gene duplication of *BBX* genes in mung bean. In the present study, no tandem duplications of *VrBBX* genes were found. Six *VrBBX* gene pairs with segmental duplication were identified in mung bean chromosomes (Fig. 6). These results indicate that segmental duplications were the main expansion pattern of mung bean *BBX* gene family members. The same potential mechanism of gene family evolution has also been identified in the NAC gene family in mung bean [41]. Previous studies have reported that tandem duplication often occurs in large and rapidly evolving gene families, while segmental duplication usually occurs in slowly evolving gene families [42]. The present results indicated that the mung bean *BBX* gene family had slow evolutionary characteristics. Furthermore, the Ka/Ks ratios of all duplicated *VrBBX* gene pairs were less than 1, except for *VrBBX1/VrBBX13*, indicating that most of the duplicated *VrBBXs* experienced purifying selective pressure (Table 2). Since purifying selection limits gene differentiation, duplicated *VrBBX* genes might retain similar functions after replication [43].

The intron-exon pattern bears the imprint of gene family evolution [44]. In the present research, the exon number of *VrBBX* genes ranged from 1 to 7, which showed similar genetic diversity to other species, such as tomato [30] and pear [31]. The different splicing patterns of exons and introns might be meaningful for the evolution of the *VrBBX* gene. Additionally, we investigated the conserved motifs of VrBBXs. Each group had several common motifs, while some groups had special motifs (Fig. 7b). The different motif compositions in each group might indicate the functional diversity of VrBBXs, and the same motif in the same group might indicate the functional synergy of VrBBXs [45]. In conclusion, the gene structures and motif distributions of VrBBXs were highly conserved in each group, supporting the close evolutionary relationships and group classifications (Fig. 7).

Transcriptional regulation of stress-responsive genes is an important part of plant stress responses. Transcription factors are potential activators or repressors that control the expression of gene clusters by binding the *cis*-acting element in the promoter regions of target genes [32]. In this study, the analysis of *VrBBX* promoter regions revealed a series of frequent occurrences of *cis*-acting elements corresponding to abiotic stress (Fig. 8), indicating their potential functions in abiotic stress. For example, the promoters of *VrBBX5* and *VrBBX10* contained drought inducibility-responsive element and the relative expression levels of *VrBBX5* and *VrBBX10* were nearly 8-fold higher than that of the control (0 h) under PEG stress (Figs. 8 and 10). To further verify the involvement of the *VrBBX* genes in the regulation of abiotic stress, the expression analysis based on the publicly available RNA sequence data and qRT-PCR were conducted. The present results suggest that the majority of *VrBBX* genes were induced or repressed to varying degrees depending on stress treatments. According to their expression changes under stress, *VrBBX10*, *VrBBX5* and *VrBBX12* can be further studied as candidate genes in response to ABA, NaCl and PEG, respectively. The expression of *VrBBX1* and *VrBBX16* was upregulated by ABA and PEG treatments but downregulated under NaCl treatment, suggesting that *VrBBX1* and *VrBBX16* might have different mechanisms to maintain protection against various abiotic signals [33]. In particular, the expression of *VrBBX5*, -*10* and -*12* was upregulated under three abiotic stresses (Fig. 10), indicating that these *VrBBX* genes may play a key role in response to multiple abiotic stress networks [33]. Previous studies have shown that the *BBX* genes in the same group might perform similar functions. For example, the *Arabidopsis BBX* genes in group I (such as *AtCO* and *AtCOL*) were mostly associated with photoperiod or photoperiod-regulated flowering [46, 47], while the majority of the *BBX* genes in group IV, including *AtBBX18*, *AtBBX19*, *AtBBX21*, *AtBBX22*, *AtBBX24* and *AtBBX25*, were related to photomorphogenesis [13, 48, 49]. The present study showed that *VrBBX1* and *VrBBX16* in group III, and *VrBBX3* and *VrBBX21* in group II showed similar expression profiles, indicating that they may have similar functions in responding to abiotic stress. The expression characterization of *VrBBX* genes in response to abiotic stress will greatly improve our understanding of the functions of abiotic stress signaling pathways.

## Conclusions

In this study, 23 *VrBBX* genes were identified in mung bean, and systematic and comprehensive analyses of the *VrBBX* gene family were performed, including conserved domain, phylogenetic tree, duplication event and expansion pattern, gene structure, conserved motif, chromosome location, *cis*-acting elements in promoters and expression patterns. The *VrBBX* genes were divided into five groups, namely I (three genes), II (six genes), III (four genes), IV (eight genes), V (two genes), which were supported by conserved domain analysis. Gene duplication analysis suggested that segmental duplication was the main expansion pattern of mung bean *BBX* genes. Numerous *cis*-acting elements were found in the *VrBBX* promoters, indicating that *VrBBX* genes are involved in complex regulatory networks that control development and responses to abiotic stress. The expression profiles of *VrBBX* genes showed that most *VrBBX* genes were responsive to ABA, PEG and NaCl treatments. Therefore, genome-wide analysis of the *VrBBX* gene family will provide a solid basis for functional analyses of *VrBBX* genes, and further study of several *VrBBX* genes is currently underway to understand their biological functions. Detailed knowledge of stress-responsive *VrBBXs* in mung bean will be a valuable resource for future molecular breeding in legumes.

### Methods

#### Identification of *BBX* gene family members in mung bean

Two methods were used to identify the *BBX* genes in mung bean. First, we used the Basic Local Alignment Search Tool (BLAST) to search for potential BBXs in the *V. radiata* genome using *Arabidopsis* BBX protein sequences as queries, and the cutoff E-value was 0.001. Second, the HMM of the B-box (pfam00643) was used to query the *V. radiata* whole-genome protein database [50]. The *V. radiata* genome database (genome assembly: Vradiata_ver6) was downloaded from EnsemblPlants (http://plants.ensembl.org/index.html). Each potential candidate sequence was verified using the SMART (http://smart.embl.de/) [51], InterProscan (http://www.ebi.ac.uk/Tools/pfa/iprscan/) [52] and Pfam (http://pfam.xfam.org/) databases for the presence of a B-box. The MW and pI of the identified VrBBXs were calculated using online ProtParam (http://web.expasy.org/protparam/) [53].

### Phylogenetic analysis and sequence alignment

The ClustalW program was used to align BBX protein sequences [54], and the phylogenetic tree was built using the MEGA 7 program with the neighbor-joining method and 1000 replicate iterations [55]. Domains were identified with SMART, InterProScan and pfam programs. The WebLogo website (http://weblogo.berkeley.edu/logo.cgi) was used to construct sequence logos of conserved domains [56]. The conserved motifs of VrBBX proteins were identified using the MEME program (http://memesuite.org/tools/meme).

### Gene structure, chromosomal location, and duplication analysis

The exon and intron locations of the *VrBBX* genes were obtained according to the mung bean genome annotation file, and a map of the *VrBBX* gene structure was generated using the GSDS website (http://gsds.cbi.pku.edu.cn/) [57]. The chromosome location image was generated with MapInspect software according to the physical positions of the *VrBBX* genes on the chromosomes or scaffolds. MCScanX software (http://chibba.pgml.uga.edu/mcscan2/) [58] was used to identify duplications of *VrBBX* genes with default parameters, and the relationship was plotted with Circos software [59]. Calculator 2.0 software [60] was used to estimate the Ka and Ks of the gene duplication pairs, and the selective pressure was calculated using the Ka/Ks ratio. The approximate date of the duplication event that occurred in the mung bean was estimated using the Formula: T = Ks/2λ × 10^–6^ Mya (λ = 6.5 × 10^− 9^) [61].

### Cis-regulatory elements and expression pattern of transcriptome analysis

The genomic DNA sequence approximately 1.5 kb upstream of the transcriptional start sites was used to analyze the *cis*-acting elements. The PLACE website (http://www.dna.affrc.go.jp/PLACE/signalscan.html) [62] was used to identify the *cis*-acting elements involved in abiotic stress and hormone responses. To gain insight into the expression patterns of *VrBBXs* genes under drought stress, RNA-Seq data (BioProject: PRJNA764584) was obtained from SRA-NCBI (https://www.ncbi.nlm.nih.gov/sra) [63]. Paired-end clean reads were downloaded and aligned to the reference genome using bowtie2. Differential expression fold change of genes was calculated by comparing the treatment with the control. The genes with false discovery rate (FDR)<0.05, Log_2_FC>1 and Log_2_FC<-1 were considered to be significantly differential expression. The heatmaps were made with the mean value of Log_2_FC in expression of *VrBBX*-regulated transcript.

### Plant materials

Ten mung bean seeds (VC1973A) were sown in a pot (diameter = 22 cm) filled with nutrient soil and cultured in a growth chamber at 24 °C with a 16 h/8 h light/dark cycle. After the first trifoliolate leaf was fully expanded, 20% PEG-6000, 100 mM NaCl and 100 µM ABA solution were poured onto seedling. Leaves were collected after treatment at 0, 4, 12 and 24 h. Three biological replications were conducted for each sample, and each replicate included three to four plants. All collected samples were stored at − 80 °C for RNA extraction and gene expression analysis.

### Expression profile analysis of *VrBBX* genes under ABA, PEG and NaCl treatments

The total RNA from leaves was isolated using TRIzol reagent (Invitrogen, Carlsbad, CA, USA), and the RNA concentration and quality were measured using NanoDrop Spectrophotometer (Thermo Fisher Scientifc, Waltham, MA, USA), with 260/280 ≥ 1.8 and 260/230 ≥ 2.0. First-strand cDNA was synthesized using a SuperScript™ III Reverse Transcriptase Kit (Invitrogen) according to the manufacturer’s instructions. The resulting cDNA was used for qRT-PCR analysis using SYBR Green PCR mix (Takara, Tokyo, Japan) with the ABI 7500 Real-Time PCR System. The PCR conditions were as follows: 95 °C for 2 min followed by 45 cycles of 95 °C for 10 s and 60 °C for 1 min. Additional file 7 lists the primer sequences used for qRT-PCR. The relative expression levels of *VrBBX* genes were determined using the 2^–ΔΔCT^ method with the *VrACTIN*3 gene (*Vradi03g00210*) serving as an endogenous control. The expression patterns of the *VrBBX* genes were identified by three independent biological replicates.

### Electronic supplementary material

Below is the link to the electronic supplementary material.

Supplementary Material 1

Supplementary Material 2

Supplementary Material 3

Supplementary Material 4

Supplementary Material 5

Supplementary Material 6

Supplementary Material 7

## Data Availability

The *Arabidopsis* BBX protein sequences were collected from the *Arabidopsis* information source database (http://www.arabidopsis.org). The BBX protein sequences of mung bean and soybean were obtained from EnsemblPlants (http://plants.ensembl.org/index.html). The genome sequences of mung bean, *Arabidopsis*, soybean and rice were downloaded from EnsemblPlants. The datasets used and analyzed during the study are available from the corresponding author on reasonable request.

## Abbreviations

BBX: B-box
CCT: CONSTANS, CO-like and TOC1
ABA: Abscisic acid
PEG: Polyethylene glycol
NaCl: Sodium chloride
B1: B-box1
B2: B-box2
CO: CONSTANS
FT: Flowering Locus T
ROS: Reactive oxygen species
SOD: Superoxide dismutase, APX: Ascorbate peroxidase
GST: Glutathione s-transferase
Mya: Million years
HMM: Hidden Markov Model
CDS: Coding sequence length, MW: Molecular weight
pI: Isoelectric point
UTR: Untranslated regions
MeJA: Methyl jasmonate
BLAST: Basic Local Alignment Search Tool
SMART: Simple Modular Architecture Research Tool
MEGA: Molecular Evolutionary Genetics Analysis
NJ: Neighbor-joining
MEME: Multiple EM for Motif Elicitation
Ka: Non-synonymous substitution rate
Ks: synonymous substitution rate
qRT‒PCR: Quantitative real-time PCR

## References

[1] Kiełbowicz-Matuk A (2012). Involvement of plant C2H2-type zinc finger transcription factors in stress responses. Plant Sci.

[2] Crocco CD, Botto JF (2013). BBX proteins in green plants: insights into their evolution, structure, feature and functional diversification. Gene.

[3] Khanna R, Kronmiller B, Maszle DR, Coupland G, Holm M, Mizuno T, Wu S-H (2009). The *Arabidopsis* B-Box Zinc Finger Family. Plant Cell.

[4] Huang J, Zhao X, Weng X, Wang L, Xie W (2012). The Rice B-Box Zinc Finger Gene Family: genomic identification, characterization, expression profiling and Diurnal Analysis. PLoS ONE.

[5] Gendron JM, Pruneda-Paz JL, Doherty CJ, Gross AM, Kang SE, Kay SA (2012). *Arabidopsis* circadian clock protein, TOC1, is a DNA-binding transcription factor. Proc Natl Acad Sci U S A.

[6] Robson F, Costa MM, Hepworth SR, Vizir I, Piñeiro M, Reeves PH, Putterill J, Coupland G (2001). Functional importance of conserved domains in the flowering-time gene *CONSTANS* demonstrated by analysis of mutant alleles and transgenic plants. Plant J.

[7] Cao J, Yuan J, Zhang Y, Chen C, Zhang B, Shi X, Niu R, Lin F (2023). Multi-layered roles of BBX proteins in plant growth and development. Stress Biol.

[8] Cheng XF, Wang ZY (2005). Overexpression of *COL9*, a *CONSTANS-LIKE* gene, delays flowering by reducing expression of *CO* and *FT* in *Arabidopsis thaliana*. Plant J.

[9] Tripathi P, Carvallo M, Hamilton EE, Preuss S, Kay SA, Arabidopsis (2017). B-BOX32 interacts with CONSTANS-LIKE3 to regulate flowering. Proc Natl Acad Sci U S A.

[10] Datta S, Hettiarachchi GH, Deng XW, Holm M, Arabidopsis (2006). CONSTANS-LIKE3 is a positive regulator of red light signaling and root growth. Plant Cell.

[11] Xu D, Jiang Y, Li J, Lin F, Holm M, Deng XW (2016). BBX21, an *Arabidopsis* B-box protein, directly activates HY5 and is targeted by COP1 for 26S proteasome-mediated degradation. Proc Natl Acad Sci U S A.

[12] Zhang X, Huai J, Shang F, Xu G, Tang W, Jing Y, Lin R (2017). A PIF1/PIF3-HY5-BBX23 transcription factor Cascade affects photomorphogenesis. Plant Physiol.

[13] Xu D, Jiang Y, Li J, Holm M, Deng XW (2018). The B-Box domain protein BBX21 promotes photomorphogenesis. Plant Physiol.

[14] Fan XY, Sun Y, Cao DM, Bai MY, Luo XM, Yang HJ, Wei CQ, Zhu SW, Sun Y, Chong K (2012). BZS1, a B-box protein, promotes photomorphogenesis downstream of both brassinosteroid and light signaling pathways. Mol Plant.

[15] Gangappa SN, Crocco CD, Johansson H, Datta S, Hettiarachchi C, Holm M, Botto JF (2013). The *Arabidopsis* B-BOX protein BBX25 interacts with HY5, negatively regulating *BBX22* expression to suppress seedling photomorphogenesis. Plant Cell.

[16] Lin F, Jiang Y, Li J, Yan T, Fan L, Liang J, Chen ZJ, Xu D, Deng XW (2018). B-BOX DOMAIN PROTEIN28 negatively regulates photomorphogenesis by repressing the activity of transcription factor HY5 and undergoes COP1-Mediated degradation. Plant Cell.

[17] Crocco CD, Holm M, Yanovsky MJ, Botto JF (2010). AtBBX21 and COP1 genetically interact in the regulation of shade avoidance. Plant J.

[18] Lippuner V, Cyert MS, Gasser CS (1996). Two classes of plant cDNA clones differentially complement yeast calcineurin mutants and increase salt tolerance of wild-type yeast. J Biol Chem.

[19] Nagaoka S, Takano T (2003). Salt tolerance-related protein STO binds to a myb transcription factor homologue and confers salt tolerance in *Arabidopsis*. J Exp Bot.

[20] Xu D, Li J, Gangappa SN, Hettiarachchi C, Lin F, Andersson MX, Jiang Y, Deng XW, Holm M (2014). Convergence of light and ABA signaling on the *ABI5* promoter. PLoS Genet.

[21] Mbambalala N, Panda SK, Vyver CVD (2021). Overexpression of *AtBBX29* improves Drought Tolerance by maintaining photosynthesis and enhancing the antioxidant and osmolyte capacity of sugarcane plants. Plant Mol Biol Rep.

[22] Xu YJ, Zhao X, Aiwaili P, Mu XY, Zhao M, Zhao J, Cheng L, Ma C, Gao JP, Hong B (2020). A zinc finger protein BBX19 interacts with ABF3 to affect drought tolerance negatively in chrysanthemum. Plant J.

[23] Liu YN, Chen H, Ping Q, Zhang ZX, Guan ZY, Fang WM, Chen SM, Chen FD, Jiang JF, Zhang F (2019). The heterologous expression of *CmBBX22* delays leaf senescence and improves drought tolerance in *Arabidopsis*. Plant Cell Rep.

[24] Yang YJ, Ma C, Xu YJ, Wei Q, Imtiaz M, Lan HB, Gao S, Cheng L, Wang MY, Fei ZJ, Hong B, Gao JP (2014). A zinc finger protein regulates flowering time and abiotic stress tolerance in Chrysanthemum by modulating Gibberellin Biosynthesis. Plant Cell.

[25] Liu X, Li R, Dai YQ, Yuan L, Sun QH, Zhang SZ, Wang XY (2019). A B-box zinc finger protein, MdBBX10, enhanced salt and drought stresses tolerance in *Arabidopsis*. Plant Mol Biol.

[26] Dai YQ, Lu Y, Zhou Z, Wang XY, Ge HJ, Sun QH (2022). B-box containing protein 1 from *Malus domestica* (MdBBX1) is involved in the abiotic stress response. Peer J.

[27] Huang SJ, Chen CH, Xu MX, Wang GB, Xu LA, Wu YQ (2021). Overexpression of Ginkgo *BBX25* enhances salt tolerance in transgenic *Populus*. Plant Physiol Biochem.

[28] Wu HF, Wang X, Cao YZ, Zhang HY, Hua R, Liu HM, Sui SZ (2021). *CpBBX19*, a B-Box transcription factor gene of Chimonanthus praecox, improves Salt and Drought Tolerance in *Arabidopsis*. Genes (Basel).

[29] Zhang H, Wang Z, Li X, Gao XR, Dai ZR, Cui YF, Zhi YH, Liu QC, Zhai H, Gao SP, Zhano N, He SZ (2022). The IbBBX24-IbTOE3-IbPRX17 module enhances abiotic stress tolerance by scavenging reactive oxygen species in sweet potato. New Phytol.

[30] Chu Z, Wang X, Li Y, Yu H, Li J, Lu Y, Li H, Ouyang B (2016). Genomic Organization, phylogenetic and expression analysis of the B-BOX Gene Family in Tomato. Front Plant Sci.

[31] Cao Y, Han Y, Meng D, Li D, Jiao C, Jin Q, Lin Y, Cai Y (2017). *B-BOX* genes: genome-wide identification, evolution and their contribution to pollen growth in pear (*Pyrus Bretschneideri* Rehd). BMC Plant Biol.

[32] Liu X, Li R, Dai YQ, Chen XW, Wang XY (2018). Genome-wide identification and expression analysis of the B-box gene family in the Apple (*Malus domestica* Borkh.) Genome. Mol Genet Genomics.

[33] Wei HR, Wang PP, Chen JQ, Li CJ, Wang YZ, Yuan YB, Fang JG, Leng XP (2020). Genome-wide identification and analysis of *B-BOX* gene family in grapevine reveal its potential functions in berry development. BMC Plant Biol.

[34] Talar U, Kiełbowicz-Matuk A, Czarnecka J, Rorat T (2017). Genome-wide survey of B-box proteins in potato (*Solanum tuberosum*)-Identification, characterization and expression patterns during diurnal cycle, etiolation and de-etiolation. PLoS ONE.

[35] Kang YJ, Kim SK, Kim MY, Lestari P, Kim KH, Ha BK, Jun TH, Hwang WJ, Lee T, Lee J (2014). Genome sequence of mungbean and insights into evolution within Vigna species. Nat Commun.

[36] Gangappa SN, Botto JF (2014). The BBX family of plant transcrip tion factors. Trends Plant Sci.

[37] Yu J, Hu S, Wang J, Wong GK, Li S, Liu B, Deng Y, Dai L, Zhou Y, Zhang X (2002). A draft sequence of the rice genome (*Oryza sativa* L. ssp. *indica*). Science.

[38] Initiative AG (2000). Analysis of the genome sequence of the flowering plant *Arabidopsis thaliana*. Nature.

[39] Kenrick P, Crane PR (1997). The origin and early evolution of plants on land. Nature.

[40] Moore RC, Purugganan MD (2003). The early stages of duplicate gene evolution. Proc Natl Acad Sci U S A.

[41] Tariq R, Hussain A, Tariq A, Khalid MHB, Khan I, Basim H, Ingvarsson PK (2022). Genome-wide analyses of the mung bean NAC gene family reveals orthologs, co-expression networking and expression profiling under abiotic and biotic stresses. BMC Plant Biol.

[42] Cannon SB, Mitra A, Baumgarten A, Young ND, May G (2004). The roles of segmental and tandem gene duplication in the evolution of large gene families in *Arabidopsis thaliana*. BMC Plant Biol.

[43] Liu W, Li W, He Q, Daud MK, Chen J, Zhu S (2014). Genome-wide survey and expression analysis of calcium-dependent protein kinase in *Gossypium Raimondii*. PLoS ONE.

[44] Lynch M (2002). Intron evolution as a population-genetic process. Proc Natl Acad Sci U S A.

[45] Liu X, Chu Z (2015). Genome-wide evolutionary characterization and analysis of bZIP transcription factors and their expression profiles in response to multiple abiotic stresses in *Brachypodium distachyon*. BMC Genomics.

[46] Ledger S, Strayer C, Ashton F, Kay SA, Putterill J (2001). Analysis of the function of two circadian-regulated *CONSTANS-LIKE* genes. Plant J.

[47] Hassidim M, Harir Y, Yakir E, Kron I, Green RM (2009). Over-expression of *CONSTANS-LIKE 5* can induce flowering in short-day grown *Arabidopsis*. Planta.

[48] Wang Q, Zeng J, Deng K, Tu X, Zhao X, Tang D, Liu X (2011). DBB1a, involved in gibberellin homeostasis, functions as a negative regulator of blue light-mediated hypocotyl elongation in *Arabidopsis*. Planta.

[49] Datta S, Johansson H, Hettiarachchi C, Irigoyen ML, Desai M, Rubio V, Holm M (2008). LZF1/SALT TOLERANCE HOMOLOG3, an *Arabidopsis* B-box protein involved in light-dependent development and gene expression, undergoes COP1-mediated ubiquitination. Plant Cell.

[50] Finn RD, Bateman A, Clements J, Coggill P, Eberhardt RY, Eddy SR, Heger A, Hetherington K, Holm L, Mistry J (2014). Pfam: the protein families database. Nucleic Acids Res.

[51] Letunic I, Doerks T, Bork P (2015). SMART: recent updates, new developments and status in 2015. Nucleic Acids Res.

[52] Zdobnov EM, Apweiler R (2001). InterProScan–an integration platform for the signature-recognition methods in InterPro. Bioinformatics.

[53] Wilkins MR, Gasteiger E, Bairoch A, Sanchez JC, Williams KL, Appel RD, Hochstrasser DF (1999). Protein identification and analysis tools in the ExPASy server. Methods Mol Biol.

[54] Thompson JD, Higgins DG, Gibson TJ (1994). CLUSTAL W: improving the sensitivity of progressive multiple sequence alignment through sequence weighting, position-specific gap penalties and weight matrix choice. Nucleic Acids Res.

[55] Kumar S, Stecher G, Tamura K (2016). MEGA7: Molecular Evolutionary Genetics Analysis Version 7.0 for bigger datasets. Mol Biol Evol.

[56] Crooks GE, Hon G, Chandonia JM, Brenner SE (2004). WebLogo: a sequence logo generator. Genome Res.

[57] Guo AY, Zhu QH, Chen X, Luo JC (2007). GSDS: a gene structure display server. Yi Chuan.

[58] Wang Y, Tang H, Debarry JD, Tan X, Li J, Wang X, Lee TH, Jin H, Marler B, Guo H (2012). MCScanX: a toolkit for detection and evolutionary analysis of gene synteny and collinearity. Nucleic Acids Res.

[59] Krzywinski M, Schein J, Birol I, Connors J, Gascoyne R, Horsman D, Jones SJ, Marra MA (2009). Circos: an information aesthetic for comparative genomics. Genome Res.

[60] Wang D, Zhang Y, Zhang Z, Zhu J, Yu J (2010). KaKs_Calculator 2.0: a toolkit incorporating gamma-series methods and sliding window strategies. Genomics.

[61] Zhou H, Qi K, Liu X, Yin H, Wang P, Chen J, Wu J, Zhang S (2016). Genome-wide identification and comparative analysis of the cation proton antiporters family in pear and four other *Rosaceae* species. Mol Genet Genomics.

[62] Lescot M, Déhais P, Thijs G, Marchal K, Moreau Y, Van de Peer Y, Rouzé P, Rombauts S (2002). PlantCARE, a database of plant *cis*-acting regulatory elements and a portal to tools for in silico analysis of promoter sequences. Nucleic Acids Res.

[63] Guo YN, Zhang SY, Ai J, Zhang PP, Yao H, Liu YF, Zhang X (2023). Transcriptomic and biochemical analyses of drought response mechanism in mung bean (*Vignaradiata* (L.) Wilczek) leaves. PLoS ONE.

