# Supplementary figures and images for: Genome-wide identification, molecular evolution and expression analysis of the B-box gene family in mung bean (Vigna radiata L.)
Source: BMC Plant Biol. 2024 Jun 12;24:532. doi: 10.1186/s12870-024-05236-9 (PMC11167828; doi:10.1186/s12870-024-05236-9)

Additional file 1. Chromosome distribution of mung bean *BBX* genes.


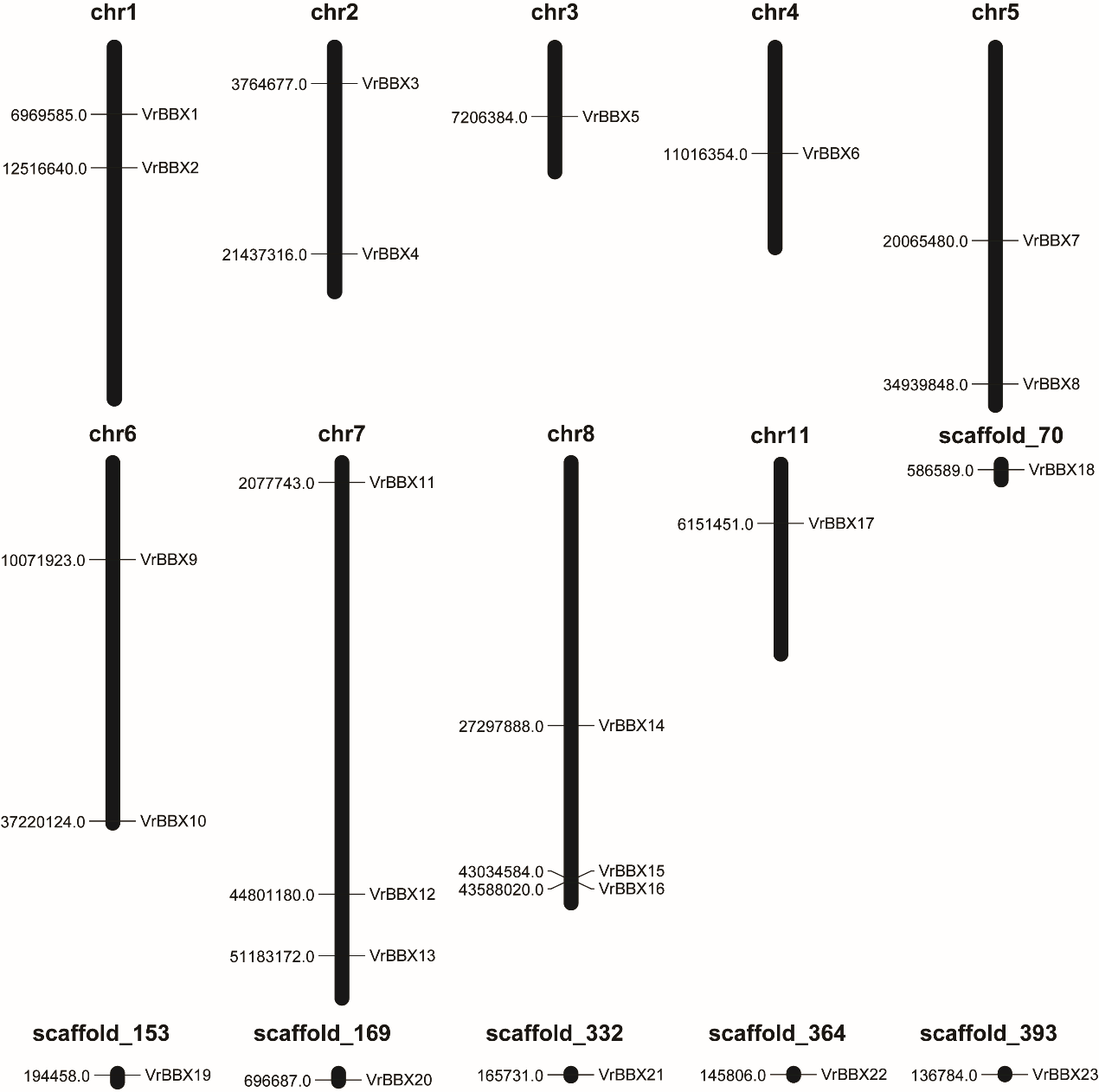

Supplement: Supplementary file 1 — Supplementary Material 1 [file 12870_2024_5236_MOESM1_ESM.docx]

Additional file 3: Multiple sequence alignments of B-box2 in VrBBXs from group I and group II.


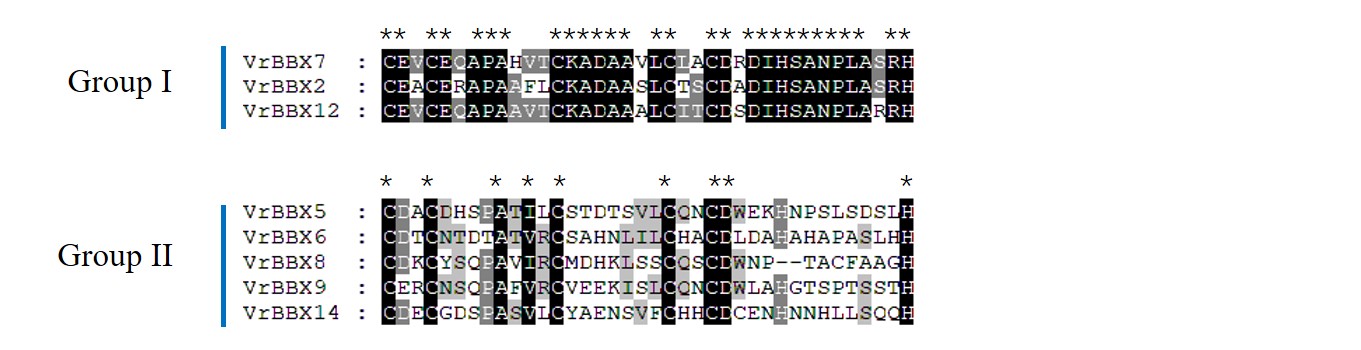

Supplement: Supplementary file 3 — Supplementary Material 3 [file 12870_2024_5236_MOESM3_ESM.docx]
