# Supplementary material for: Genome-wide identification, molecular evolution and expression analysis of the B-box gene family in mung bean (Vigna radiata L.)
Source: BMC Plant Biol. 2024 Jun 12;24:532. doi: 10.1186/s12870-024-05236-9 (PMC11167828; doi:10.1186/s12870-024-05236-9)

Additional file 5: The sequences and logos of the 7 motifs in VrBBXs.

| Motif | | Motif sequences |
| --- | --- | --- |
| Motif 1 | LLFRQRVEFPGDKPAQAENPGSQPLDPGESKRGQNQLPKLKMGEKQQNHVMPLVPTPENNADGHAKMDTKMIDLNMKP | |
| Motif 2 | LTGVRVGLEATEPGASSSSLKSDSGEKISDAKSSSISRKVSSEPQNPDFNEMFPNEGGGVEGFPPNKESFGGGYTVGNISQWPIEEFJGLNEFSQNYDYM | |
| Motif 3 | RPJNCYSGCPSAAEFSSIWGF | |
| Motif 4 | RFLATGIRVALGSNCTKGNEKGHLEPPNRNAQZVPVKVPSQQLPSFTSSWAVDDFLELTGFE | |
| Motif 5 | KKDJFLRLNYEDVISAWSSQGS | |
| Motif 6 | FGELEWLADVGJFGEQFPZEPLAAAEVPQLPVTN | |
| Motif 7 | GFTKKARTPRH | |

The logos of the 7 motifs.

Motif1


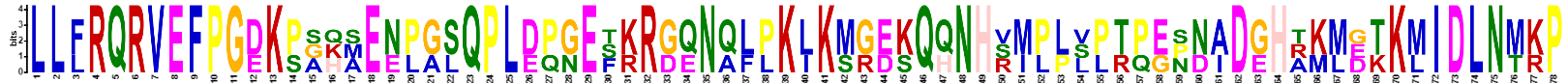


Motif2


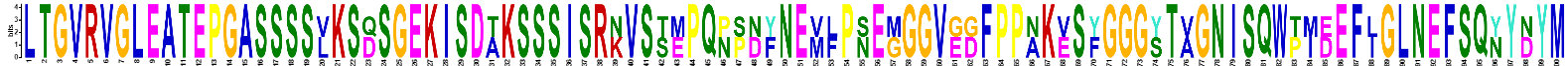


Motif3


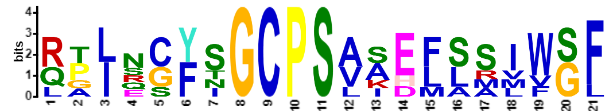


Motif4


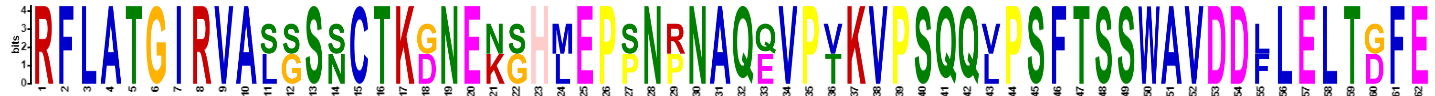


Motif5


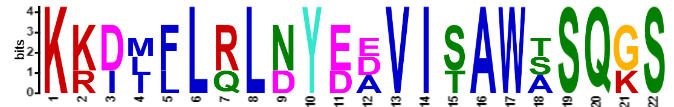


Motif6


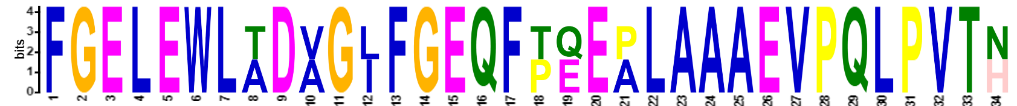


Motif7


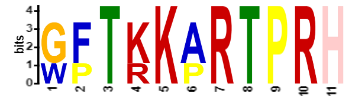

Supplement: Supplementary file 5 — Supplementary Material 5 [file 12870_2024_5236_MOESM5_ESM.docx]
