# Supplementary material for: Genome-wide identification, molecular evolution and expression analysis of the B-box gene family in mung bean (Vigna radiata L.)
Source: BMC Plant Biol. 2024 Jun 12;24:532. doi: 10.1186/s12870-024-05236-9 (PMC11167828; doi:10.1186/s12870-024-05236-9)
Supplement: Supplementary file 7 — Supplementary Material 7 [file 12870_2024_5236_MOESM7_ESM.docx]

Additional file 7: Primers used for qRT-PCR in this study.

| **Gene name** | **Forward primer** **(5’-3’)** | **Tm of Forward primer** | **Reverse primer (5’-3’)** | **Tm of Reverse primer** |
| --- | --- | --- | --- | --- |
| *VrBBX1* | ATCAAATCTCCAGCCAATGAGC | 55.8℃ | TGCTTCTTTCATTTCGTCGG | 53.4℃ |
| *VrBBX3* | GAGGCTTCTTCTTCAATGTT | 51.1℃ | TTGGCTCATCACAGTCTT | 50.6℃ |
| *VrBBX5* | GCGCGACCCTGTGACTACTGTG | 63.3℃ | GTCCGCGTGTGCTTCGAGAA | 59.5℃ |
| *VrBBX10* | GATGTTTGTCAGGAGAGAAGAG | 55.8℃ | AGTGAGAAGGAACCTATCATGC | 55.8℃ |
| *VrBBX12* | CGCCAAAGCCATGTGACT | 54.9℃ | CTTGTTGGCGCAGTGAATCTTG | 57.7℃ |
| *VrBBX16* | TACTGTGCTGCCGATGAT | 51.9℃ | GGAGGTTGTTGTTGTTGTTC | 52.6℃ |
| *VrBBX19* | AGAATCTGGCGAGGTTGGAG | 57.5℃ | TCGTGCAATTCGCGGGGTAG | 59.5℃ |
| *VrBBX21* | TGGAATGGCTTACAGATG | 55.9℃ | GTAGGACTTGGAGGTTCT | 57.5℃ |
| *VrBBX22* | GCCAGGAAGCATTAGGATA | 52.8℃ | TGACCAGAGACATAAGCATT | 50℃ |
